# Emotion regulation across psychiatric disorders

**DOI:** 10.1017/S1092852924000270

**Published:** 2024-05-02

**Authors:** Ibrahim H. Aslan, Lucy Dorey, Jon E. Grant, Samuel R. Chamberlain

**Affiliations:** a Department of Psychiatry, Faculty of Medicine, University of Southampton, Southampton, UK; b Southern Health NHS Foundation Trust, Southampton, UK; c Department of Psychiatry & Behavioral Neuroscience, University of Chicago, Chicago, IL, USA

**Keywords:** emotion regulation, impulsivity, trans-diagnostic, emotional dysregulation

## Abstract

**Objective:**

Difficulties with emotion regulation have been associated with multiple psychiatric conditions, yet little is known about how emotional dysregulation varies among these disorders. In this study, we aimed to investigate emotional regulation difficulties in young adults who gamble at least occasionally (i.e. an enriched sample), diagnosed with a range of psychiatric disorders using the validated Difficulties in Emotion Regulation Scale (DERS).

**Methods:**

A total of 543 non-treatment seeking individuals who had engaged in gambling activities on at least five occasions within the previous year, aged 18-29 were recruited from general community settings. Diagnostic assessments included the Mini International Neuropsychiatric Inventory (MINI), Minnesota Impulsive Disorders Interview (MIDI), attention-deficit/hyperactivity disorder (ADHD) World Health Organization Screening Tool Part A (ASRS v1.1), and the Structured Clinical Interview for Gambling Disorder. Emotional dysregulation was evaluated using DERS. The profile of emotional dysregulation across disorders was characterised using z-scores (those with the index disorder versus those without the index disorder).

**Results:**

Individuals with probable ADHD displayed the highest level of difficulties in emotional regulation, followed by intermittent explosive disorder, social phobia, and generalized anxiety disorder. In contrast, participants diagnosed with obsessive-compulsive disorder showed relatively lower levels of difficulties with emotional regulation.

**Conclusions:**

This study highlights the importance of recognizing emotional dysregulation as a trans-diagnostic phenomenon across psychiatric disorders. The results also reveal differing levels of emotional dysregulation across diagnoses, with potential implications for tailored treatment approaches. Despite limitations such as small sample sizes for certain disorders and limited age range, this study contributes to a broader understanding of emotional regulation's role in psychiatric conditions.

## Introduction

Emotional dysregulation, characterized by the inability to effectively manage and modulate emotions, is a prominent feature in various psychiatric disorders^1–3^. This phenomenon encompasses a range of difficulties, including difficulties in regulating emotional responses, heightened emotional reactivity and impaired emotional expression^1,2^. While emotional dysregulation has been extensively studied within individual psychiatric disorders, a comprehensive exploration of its manifestations across different diagnostic categories has been relatively limited. This study aims to address this gap by examining difficulties with emotional regulation across psychiatric disorders in one large community recruited sample, specifically focusing on individuals who gamble at least occasionally, shedding light on its trans-diagnostic nature and potential implications for treatment strategies. By focusing on people who gamble at least occasionally, this yields an enriched sample i.e. a sample of people more likely to have various mental disorders than the general population (since gambling itself is linked to many mental disorders).

Previous studies showed that maladaptive emotion regulation strategies such as avoidance, rumination and self-blame are frequently involved in the development and maintenance of various psychiatric disorders, including but not limited to mood^4^ and anxiety disorders^5^, obsessive-compulsive disorder^6^, alcohol and substance use disorders^7,8^, gambling disorder^9,10^, eating disorders^11^, and body-focused repetitive disorders^12^ (trichotillomania and skin-picking disorder); as well as in other clinically relevant syndrome such as in problematic exercise^13^, problematic smartphone use^14^ and compulsive sexual behaviour^15^. Individuals with mental health disorders often struggle to effectively recognize, regulate, and respond to emotions in a manner that aligns with the demands of their social and environmental contexts^1,3^. Furthermore, models of gambling disorder^9,10^, eating disorders^16,17^ and alcohol use disorder^18,19^ suggest that individuals who cannot effectively regulate their emotions might frequently turn to gambling, food or alcohol as emotional escape leading to negative reinforcement of unhelpful behavioural patterns.

Emotional dysregulation can lead to a range of negative outcomes, including impaired interpersonal relationships, hindered daily functioning, and increased susceptibility to comorbid disorders^20^. Furthermore, given the relationship between emotional dysregulation and impulsivity, it becomes evident that factoring in emotion regulation is essential when evaluating individuals at a heightened risk of developing an addiction^21^. Additionally, it is crucial to acknowledge the broader context of emotional dysregulation, especially its linkage to hypersexuality. Lew-Starowicz and colleagues explored the complex connection between compulsive sexual behaviour (CSB) and emotion dysregulation, shedding light on how emotional dysregulation serves as a core element in individuals suffering from CSB. The study investigated patterns of emotion dysregulation as a common clinical feature, underlying mechanisms, and target for psychological and pharmacological interventions in individuals with CSB. The findings suggest that emotional dysregulation plays a pivotal role in the onset and severity of CSB, emphasizing its relevance in both understanding and treating compulsive sexual behaviour.^15^

Recent advances in neuroimaging, neurobiology, and psychological research have provided a foundation for exploring emotional dysregulation as a cross-cutting construct. This approach allows researchers to investigate whether certain neural circuits, genetic factors, or psychological mechanisms are consistently implicated in emotional dysregulation across diverse psychiatric disorders^22–24^. Moreover, understanding the shared mechanisms underlying emotional dysregulation could pave the way for the development of interventions that target these mechanisms across multiple disorders, potentially offering more effective and personalized treatment approaches.

Despite the centrality of emotional (dys)regulation in various psychiatric conditions, research has primarily focused on understanding this phenomenon within isolated disorder-specific contexts. This compartmentalized approach may overlook the commonalities that exist across disorders, hindering our understanding of the broader patterns and mechanisms that contribute to emotional (dys)regulation. By adopting a trans-diagnostic perspective using the same recruitment techniques and methodology, we can unravel the shared underpinnings of emotional dysregulation across psychiatric disorders, potentially revealing novel insights into its aetiology and treatment strategies; this can also help to identify the relative degree of emotional dysregulation across different disorders. Therefore, this study aimed to examine difficulties in emotion regulation across a range of psychiatric disorders using the Difficulties in Emotion Regulation Scale (DERS) in a diverse, nontreatment seeking sample of young adults, in a single study setting.

## Methods

### Participants

A total of 543 participants (aged 18-29 years) were enrolled from general community settings. In this study, the criteria for participant inclusion were as follows: individuals who had engaged in gambling activities on at least five occasions within the previous year. This criterion was established as part of a larger longitudinal study that specifically focused on impulsivity in young adults. Additionally, participants needed to be available for in-person interviews. To minimize the potential influence of age-related factors on emotional regulation difficulties, a specific and limited age range was selected. The exclusion criteria for the study comprised individuals with hearing or vision impairments, as well as those lacking the capacity to comprehend the study's objectives and provide informed consent. Participants were recruited through media advertisements in the Minneapolis and Chicago metropolitan regions and were compensated with a $50 gift card. The study's design and consent statement were approved by the University of Chicago's Institutional Review Board (IRB). Prior to enrolment, potential participants were fully informed about the study's details, offering them the chance to seek clarification and address queries. Written informed consent was obtained from participants, ensuring their understanding and voluntary participation. All procedures contributing to this work comply with the ethical standards of the relevant national and institutional committees on human experimentation and with the Helsinki Declaration of 1975, as revised in 2008.

### Assessments

Demographic variables (age, biological sex at birth, gender, and highest level of education) were recorded. Several previously psychometrically validated instruments were used. Participants were evaluated for psychiatric disorders using the Mini International Neuropsychiatric Inventory (MINI)^25^ (The MINI covers a range of psychiatric disorders, including but not limited to major depressive disorder, panic disorder, agoraphobia, social anxiety disorder, OCD, PTSD, alcohol dependence, alcohol abuse, substance dependence, substance abuse, bulimia, generalized anxiety disorder (GAD), antisocial personality disorder, gambling disorder), the Minnesota Impulsive Disorders Interview (MIDI) (which screens for compulsive buying, kleptomania, trichotillomania, skin-picking disorder, pyromania, intermittent explosive disorder, compulsive sexual behaviour, and binge-eating disorder)^26,27^, the ADHD World Health Organization Screening Tool Part A (ASRS v1.1)^28^, and the Structured Clinical Interview for Gambling Disorder (SCI-GD)^29^. Those meeting WHO recommended scoring criteria for probable ADHD were referred to as ‘probable ADHD’ to recognise that this is not a formal diagnostic instrument (unlike the other instruments).

In addition to diagnostic measures, emotion (dys)regulation was assessed with the well-established Difficulties in Emotion Regulation Scale (DERS)^30^. This instrument has demonstrated high internal consistency and test-retest reliability. The DERS measures all main features of emotion regulation, encompassing aspects such as accurately identifying emotional states, responding to negative emotions with secondary negative emotions, maintaining goal-directed behaviour in the presence of negative emotions, refraining from impulsive actions during negative emotional states, attending to emotional experiences, believing in limited efficacy for emotion regulation. Responses to the 36 items of the DERS are recorded on a scale ranging from 0 to 5, corresponding to "almost never" to "almost always." The measure of interest in this study was the total DERS score, with a higher total score indicating greater difficulties regulating emotions.

### Data analysis

For analysis, only psychiatric diseases that were identified in at least 1% of the participants (i.e., at least 5 individuals per diagnosis) were considered. By calculating z-scores in relation to normative data from study participants who did not have the reference mental disorder (hereafter referred to as controls), the profile of difficulties in emotion regulation for disorders was quantified. In this context, z-scores are equivalent to Cohen's d and represent effect sizes. By convention, effect sizes of 0.3, 0.5, and 0.8 are considered small, medium, and large, respectively. Each analysis compared all individuals with the given mental disorder of interest (irrespective of whether they had other disorders or not) to controls (people without the given mental disorder of interest, whether or not they had other morbidities). This allowed for a thorough examination of the emotional regulation difficulties associated with individual psychiatric disorders, even in the presence of comorbidity, in a naturalistic fashion.

## Results

A total of 543 young adults [mean (standard deviation) age = 22.3 (3.6) years; n = 192 female (35.4%)] were enrolled. Of the 543 participants, 482 (88.8%) had completed some college education or higher. The numbers [%] of participants with each disorder of interest were as follows (Table 1): major depressive disorder 12 [2.2%], panic disorder 7 [1.3%], agoraphobia 24 [4.4%], social anxiety disorder 23 [4.2%], OCD 12 [2.2%], PTSD 6 [1.1%], alcohol dependence 76 [14.0%], alcohol abuse 70 [13.0%], substance dependence 45 [8.3%], substance abuse 41 [7.6%], bulimia 10 [1.8%], generalized anxiety disorder (GAD) 25 [4.6%], antisocial personality disorder 29 [5.3%], probable ADHD 30 [7.0%], intermittent explosive disorder 10 [2.1%], gambling disorder 94 [19.3%], compulsive sexual disorder 14 [2.9%], compulsive buying disorder 23 [4.7%], and binge-eating disorder 7 [1.4%]. Note that percentages do not always reflect a denominator of 543 cases as this refers to percentage of those who completed DERS (i.e., for whom data were available).

**Figure 1:**
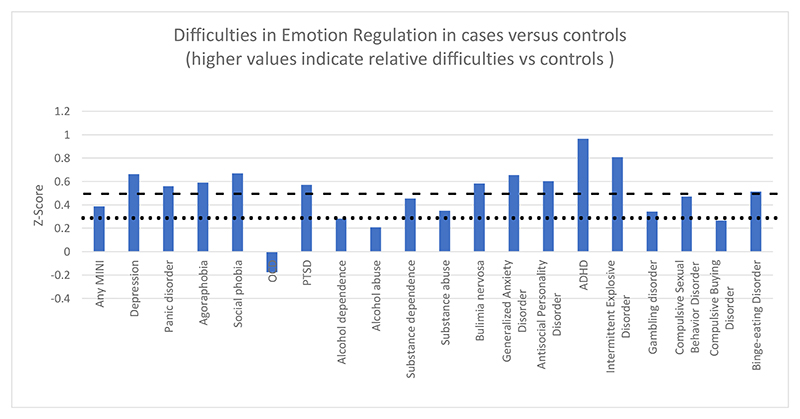
Profile of difficulties in emotion regulation across the range of mental health conditions. The panel shows z-scores for DERS total score in patient groups vs controls. The dotted lines indicate the threshold for at least small effect size deficit (z-score ≥ 0.3), and the dashed lines show the threshold for at least medium effect size deficit (z-score ≥ 0.5), vs controls. The bar ‘Any MINI’ shows the Z-Score for participants who had ANY mental disorder(s) as compared to those who had none, as a visual reference point.

Figure 1 illustrates the extent of difficulties in emotion regulation across different psychiatric disorders, as indicated by the Z-scores. Participants meeting criteria for probable ADHD (Z-score = 0.966) exhibited the highest Z-score, indicating greater difficulties in emotional regulation in this group. This was followed by intermittent explosive disorder (Z-score: 0.809), social phobia (Z-score: 0.668), depression (Z-score: 0.662), generalized anxiety disorder (Z-score: 0.656), antisocial personality disorder (Z-score: 0.603), agoraphobia (Z-score: 0.592), bulimia nervosa (Z-score: 0.583), PTSD (Z-score: 0.571), panic disorder (Z-score: 0.559), binge eating disorder (Z-score: 0.514), compulsive sexual behaviour disorder (Z-score: 0.470), any MINI (Z-score: 0.387), substance abuse (Z-score: 0.349), gambling disorder (0.343), alcohol dependence (Z-score: 0.281), compulsive buying disorder (Z-score: 0.266) and alcohol abuse (Z-score: 0.209) reflecting notable levels of emotional dysregulation in these conditions. On the other hand, participants diagnosed with OCD (Z-score = -0.177) displayed a lower Z-score than their respective control group, suggesting comparatively lower levels of difficulties in emotional regulation in this group. Disorders such as depression, panic disorder, agoraphobia and PTSD showed moderate Z-scores, indicating a moderate degree of difficulties in emotional regulation.

## Discussion

This study introduces a comprehensive evaluation of difficulties in emotional regulation across a diverse range of psychiatric disorders using the Difficulties in Emotion Regulation Scale (DERS). The findings reveal differential patterns of difficulties in emotional regulation, with some disorders displaying more pronounced difficulties in emotion regulation than others. Notably, ADHD emerges as a disorder characterized by substantial difficulties in emotional regulation, as evidenced by the highest Z-score observed of all the psychiatric conditions that were examined. This observation aligns with previous literature indicating a link between ADHD and emotional dysregulation^31^. The heightened emotional dysregulation in anxiety-related disorders aligns with the well-established connection between anxiety and emotional reactivity^32,33^.

Similarly, individuals with compulsive sexual behaviour disorder demonstrate distinctive patterns of emotional dysregulation. The literature on hypersexuality suggests a crucial interplay between emotion dysregulation and compulsive sexual behaviour^15,34^. Those affected by compulsive sexual behaviour often struggle with managing emotional states, and sexual arousal and engagement may serve as maladaptive coping mechanisms to regulate negative mood states. Our findings further support and extend this literature, revealing heightened emotional dysregulation within individuals with compulsive sexual behaviour disorder. This emphasizes the intricate relationship between emotional dysregulation and compulsive sexual behaviour, contributing to our understanding of this disorder within the broader spectrum of emotional regulation difficulties.

Personality disorders, too, have links with emotional dysregulation^20,35^. Previous research has illuminated the association between antisocial personality traits and challenges in effectively modulating and responding to emotions. Individuals with antisocial personality disorder may exhibit impulsivity, a characteristic linked to emotional dysregulation, further emphasizing the relevance of considering emotion regulation difficulties within this diagnostic category^36,37^. Our study aligns with existing literature, indicating that individuals with antisocial personality disorder indeed manifest heightened emotional dysregulation. This supports the notion that exploring emotion regulation difficulties within this diagnostic category is crucial for a comprehensive understanding of the disorder.

In contrast to the above conditions, participants diagnosed with OCD showed relatively lower levels of difficulties in emotional regulation, in fact relatively less than controls with small effect size. Earlier studies highlighted the association between OCD severity and challenges in accepting and tolerating negative emotions, aligning with the cognitive model of OCD by Salkovskis (1985)^38–40^. Similarly, studies on obsessive-compulsive personality disorder reported opposite directions in the link between the Difficulties in Emotion Regulation Scale (DERS) and obsessive-compulsive symptoms^41,42^. This discrepancy raises intriguing questions about the nuanced relationship between emotional dysregulation and obsessive-compulsive symptoms. Such discrepancy may reflect the unique profile associated with OCD, which is linked to a variety of high-order executive dysfunctions whereas perhaps emotional processing is relatively spared. It is also possible that specific OCD subtypes may exhibit different levels of difficulties in emotion regulation. These results highlight the relevance of assessing emotional dysregulation across various psychiatric diagnoses and provide insights into potential areas of focus for therapeutic interventions.

While this study is one of the first to present the profile of emotional regulation across a range of mental health disorders within a single study setting, there are several important limitations that warrant consideration. Firstly, the participant sample consisted of individuals who had engaged in gambling activities on at least five occasions within the previous year and were not actively seeking treatment, potentially limiting the applicability of the findings to clinical populations or alternative settings. At the same time, it is worth noting of course that gambling is commonplace in most countries across the globe (e.g. in the UK, around half of the adult population gambles to some degree) (source: Gambling Commission, UK). Secondly, the study's design did not incorporate controls for confounding variables that could be differentially associated with various disorders (e.g., rates of depressive symptoms) and had relatively small sample sizes for some of the disorders of interest. Relatedly, we did not control for comorbidities since the sample sizes would have been too small to facilitate this (given that comorbidity is common per disorder). It is arguably more important to consider whether affected individuals experience relative difficulties in emotional regulation in vivo, as opposed to “once comorbidities are controlled for” since the latter then would potentially underrepresent the actual problems experienced by those individuals. Similarly, we could not examine the potential role of other variables such as age, gender distribution, duration of illness, and treatments received, due to the relatively small cell sizes. For the same reason, we did not describe each disorder’s characteristics in more detail. Though the current study involved a nontreatment seeking sample, some individuals would have been receiving previously established treatments and this information (including duration of any such treatments) was not available nor could it have been analysed due to the sample sizes. Additionally, although we kept the age range narrow to reduce age effects on DERS scores, the age range of participants in this study may influence the generalizability of findings. Furthermore, these results emerged from a sample of young adults with a relatively short duration of illness for most of these disorders and therefore these findings may differ in adults with a long duration of illness (and/or duration of untreated illness). Ideally future work would use a similar “single study” approach to look at different aspects of DERS across different disorders in a much larger sample, with a wider age range, to validate these findings and ensure they are reproducible. Lastly we used clinical instruments that were previously validated, but did not conduct further psychometric validation (since this was outside the scope of this study).

## Conclusion

In conclusion, this study sheds light on the extent of emotional dysregulation within a diverse range of psychiatric disorders. The results reveal differing patterns of emotional dysregulation across diagnoses, with probable ADHD demonstrating the highest levels of emotional dysregulation and OCD showing comparatively low levels. These findings emphasize the importance of recognizing emotional dysregulation as a trans-diagnostic feature that requires tailored treatment approaches. Future research endeavours should employ longitudinal designs and explore underlying mechanisms to enhance our understanding of emotional regulation's role in psychiatric disorders.

## Figures and Tables

**Table 1:** 

Numbers of participants	Without Disorder	With Disorder	%
	N	N	%
Any MINI	342	201	37.0
Depression	528	12	2.2
Panic disorder	525	7	1.3
Agoraphobia	519	24	4.4
Social phobia	520	23	4.2
OCD	531	12	2.2
PTSD	537	6	1.1
Alcohol dependence	467	76	14.0
Alcohol abuse	470	70	13.0
Substance dependence	498	45	8.3
Substance abuse	502	41	7.6
Bulimia nervosa	531	10	1.8
Generalized Anxiety Disorder	518	25	4.6
Antisocial Personality Disorder	514	29	5.3
ADHD	401	30	7.0
Intermittent Explosive Disorder	475	10	2.1
Gambling disorder	394	94	19.3
Compulsive Sexual Behaviour Disorder	474	14	2.9
Compulsive Buying Disorder	464	23	4.7
Binge-Eating Disorder	480	7	1.4
